# Training at maximal power in resisted sprinting: Optimal load determination methodology and pilot results in team sport athletes

**DOI:** 10.1371/journal.pone.0195477

**Published:** 2018-04-11

**Authors:** Matt R. Cross, Johan Lahti, Scott R. Brown, Mehdi Chedati, Pedro Jimenez-Reyes, Pierre Samozino, Ola Eriksrud, Jean-Benoit Morin

**Affiliations:** 1 Université Savoie Mont Blanc, Laboratoire Interuniversitaire de Biologie de la Motricité, EA, Chambéry, France; 2 Département Scientifique et Sportif, Fédération Française de Ski, Annecy, France; 3 Sports Performance Research Institute New Zealand, Auckland University of Technology, Auckland, New Zealand; 4 Université Côte d’Azur, LAMHESS, Nice, France; 5 Faculty of Sports and Health Sciences, University of Jyväskylä, Jyväskylä, Finland; 6 Neuromuscular and Rehabilitation Robotics Laboratory (NeuRRo Lab), Department of Physical Medicine and Rehabilitation, University of Michigan Medical School, Ann Arbor, MI, United States of America; 7 Faculty of Physical Sciences and Sport, Catholic University of San Antonio, Murcia, Spain; 8 Department of Physical Performance, Norwegian School of Sports Science, Oslo, Norway; Norwegian University of Science and Technology, NORWAY

## Abstract

**Aims:**

In the current study we investigated the effects of resisted sprint training on sprinting performance and underlying mechanical parameters (force-velocity-power profile) based on two different training protocols: (i) loads that represented maximum power output (*L*_opt_) and a 50% decrease in maximum unresisted sprinting velocity and (ii) lighter loads that represented a 10% decrease in maximum unresisted sprinting velocity, as drawn from previous research (*L*_10_).

**Methods:**

Soccer [*n* = 15 male] and rugby [*n* = 21; 9 male and 12 female] club-level athletes were individually assessed for horizontal force-velocity and load-velocity profiles using a battery of resisted sprints, sled or robotic resistance respectively. Athletes then performed a 12-session resisted (10 × 20-m; and pre- post-profiling) sprint training intervention following the *L*_10_ or *L*_opt_ protocol.

**Results:**

Both *L*_10_ and *L*_opt_ training protocols had minor effects on sprinting performance (average of -1.4 to -2.3% split-times respectively), and provided trivial, small and unclear changes in mechanical sprinting parameters. Unexpectedly, *L*_opt_ impacted velocity dominant variables to a greater degree than *L*_10_ (trivial benefit in maximum velocity; small increase in slope of the force-velocity relationship), while *L*_10_ improved force and power dominant metrics (trivial benefit in maximal power; small benefit in maximal effectiveness of ground force orientation).

**Conclusions:**

Both resisted-sprint training protocols were likely to improve performance after a short training intervention in already sprint trained athletes. However, widely varied individualised results indicated that adaptations may be dependent on pre-training force-velocity characteristics.

## Introduction

Many popular sporting codes feature some derivative of ‘sprinting’ as a central tenant of performance. This ranges from sports where sprinting ability is the sole measure of success, such as the 100-m dash, to sports requiring athletes to sprint while moving an external mass, such as the bobsled. The relationship between sprinting acceleration and practical on-field success is not synonymous across all sporting disciplines. For example, a rugby player has little need for the same ability to generate high levels of velocity that are commonly witnessed in 200-m sprinters. In contrast, a 200-m sprinter is typically much lighter than the average rugby player, and never needs to partake in recurrent low-velocity collisions and driving forward with external mass. As such, there are distinct bands of force and velocity capacities specific to individuals, and perhaps sporting codes, that would benefit from understanding if we are to maximize the transfer between training and practical performance [1–3].

**Fig 1:**
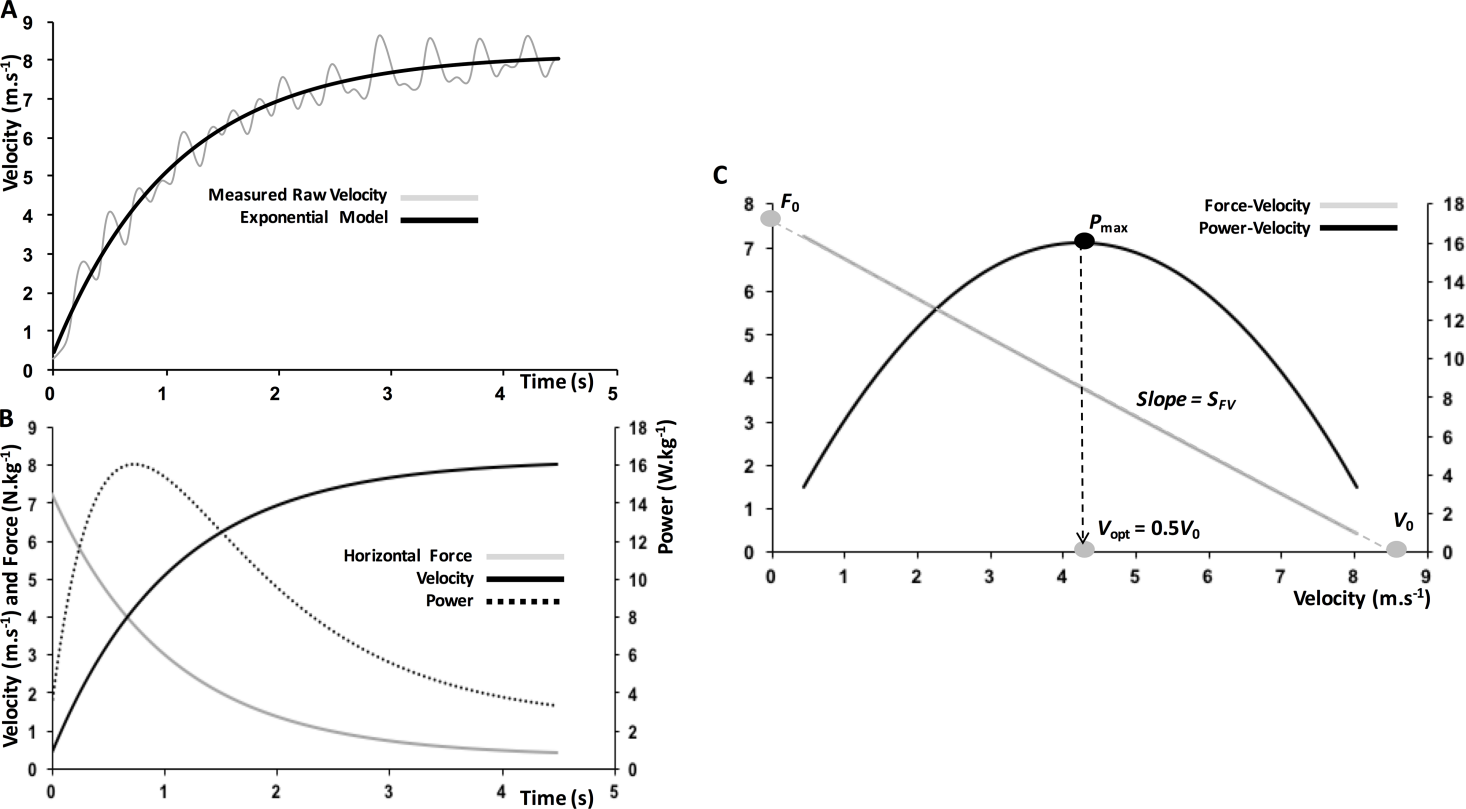
**A.** Running velocity measured with the 1080 Sprint device during a 30 m sprint acceleration, and fitted with a mono-exponential. Analyzed data was backward-extrapolated to 0 s using the subsequent equation fit. **B.** Force and power outputs in the horizontal direction are then computed from center of mass mechanics based on the methods of [13]. **C.** Force-velocity and power-velocity relationships are plotted based on the data presented in B. and used to compute maximal theoretical force *F*_0_, velocity *v*_0_, maximal power *P*_max_ and the corresponding optimal velocity *v*_opt_ = 0.5.*v*_0_ [14]. Finally, the slope of the force-velocity relationship (*S*_Fv_) indicates the force-velocity profile of the athlete (data for a 1.73 m, 95-kg rugby player). Note that the exact same procedures were used with the soccer players, except that the initial raw velocity data were recorded using a radar gun (as in Samozino et al. 2016).

During sprint acceleration, as with many other maximal movements, the mechanical ability to generate force under a range of velocities can be measured and expressed in the force-velocity (Fv) relationship. The integral of these two variables explains the ability of the athlete to express and maximize power, and has been of interest in enhancing performance in a range of movements and disciplines [4–6]. During multi-joint accelerative movements, the relationship between external force production and velocity is typically fitted with linear regressions, with power-velocity being parabolic and fitted using 2^nd^ or 3^rd^ order polynomial relationships [7]. The intercepts of this relationship represent the maximum capacity of the neuromuscular system; the force production theoretically possible in the absence of velocity (*F*_0_), and the maximum theoretical velocity until which horizontal force can be produced (*v*_0_). Maximal power (*P*_max_) is determined as the optimal combination of Fv capacities, existing in the centre of the Fv relationship as the peak of the power-velocity (*P*_v_) curve (for illustration, see Fig 1C). In the case of sprinting, these relationships represent the diverse abilities of force production at a range of velocities in a *horizontal* direction. The comparison of these variables, and the slope of the linear regression between them (*S*_Fv_), can provide insight into a number of factors underlying performance [8]. For example, Fv profiles have been shown to differ between rugby players [9] and may differentiate athletes who have suffered hamstring injury [10, 11]. In jumping, an ‘optimal profile’ can be calculated that, if attained, will maximize jump height for a maintained level of *P*_max_ *ceteris paribus* [1, 2, 12].

Fv characteristics are typically assessed using either a multiple trial method, comprising several resisted trials performed against a range of resistance, or a single trial method, comprising a single accelerative bout [7]. Both methods have been shown to provide linear Fv profiles in field testing conditions using widely accessible tools [13–15]. Most research in sprint running has used a single trial method, due to an ability to represent the mechanical output expressed during a ‘free’ (i.e. unresisted) sprinting acceleration. Samozino et al. [13] recently validated a method of profiling full sprinting acceleration phases using input data accessible to most coaches (distance- or velocity-time data of sufficient frequency). While this method provides a simple means of gaining insight into the mechanical abilities expressed by an individual, it does not yet provide a means of qualifying training goals and loading parameters [8]. Implementing a multiple trial method [14] is much more taxing on the individual, but can provide insight into loading parameters that may be of value in training implementation. This method has been recently utilized in overground resisted sled sprinting utilizing known air and sled-friction coefficients [16] and instantaneous maximum velocity (sports radar identical to that validated for the single-trial method [13]). In fact, the multiple trial method provides similar Fv data to that determined from a single sprint, and could be practically used to select loading parameters that model the external mechanical characteristics experienced during sprint phases [15]. Unfortunately, a clear practical problem with the multiple trial method is a need for distinct knowledge of the complex friction characteristics at play [16]. While computing friction coefficients is necessary in quantifying horizontal force production during resisted sprints, and complex experimental designs are needed in research of this sort, it may be needed to meet the aims of practitioners only interested in quantifying accurate training load. As will be described in this paper, the advent of the single trial method [13, 15] makes it possible to determine the individual optimal load without the need to directly quantify friction coefficients.

Members of this research group recently published a study comparing the Fv data determined from trials of external loading to that of a single unloaded sprint, and found that resisted sprint loads could feasibly model the external mechanical characteristics experienced during sprint phases, albeit with some error [15]; the broad interpretation of this finding being that training in targeted conditions may transfer to effective changes in *specific* force and velocity capacities, and concomitant changes in accelerative performance. As such, we believe the most interesting factor regarding the Fv relationship when assessed during resisted sled sprinting is the magnitude of loading corresponding to the various sections of the spectrum. A specific example are the loading conditions corresponding to *P*_max_ (i.e. ‘optimal loading *L*_opt_’ at the optimal running velocity [*v*_opt_, Fig 1C]), which have shown to to constitute a resisted-sled load of up to 96% of body-mass (~50% decrement in maximum velocity) [14]. Theoretically, training using such a load may result in increased ability to produce *P*_max_, and a practical increase in ability to transfer force throughout the sprinting phases (i.e. an increase in both force and velocity capacities) [8]. While these theories are sound, and not novel with regards to the wider circle of resistance training [8, 17], this concept is scarce in the narrative of the sprint training literature and has not been tested experimentally.

Resisted sprinting has been implemented as a method of overloading capacities specific to sprinting acceleration performance [18]. However until recently few researchers have exceeded relatively light loading parameters (e.g. approximately ~10% velocity decrement) for fear of creating dissimilar conditions to unresisted sprinting resulting in negative adaptations (e.g. slower running velocity and/or altered running technique) [19–21]. This restriction in approaches has resulted in a general lack of knowledge around the effects of training at magnitudes that would constitute anything other than a ‘velocity’ based external training stimuli (based on the proposed model). There is some preliminary evidence to suggest that training using much heavier resistances may be beneficial for accelerative performance [22, 23], however more evidence is required, especially with *accurately* and *individually* determined *L*_opt_.

When it comes to manipulating the constraints of sprinting determinants, there is no real consensus on the method of implementing resisted sprint training. The aim of this pilot study in trained rugby and soccer players was to compare the effects on sprint performance and mechanical outputs of a resisted sprint training program centred on the individual *L*_opt_ for *P*_max_ versus a control, lighter load associated with a decrement of 10% in maximal running velocity (*L*_10_). We hypothesise that resisted sprint training in the individual optimal loading condition (i.e. in the Fv zone of *P*_max_ production) would result in greater improvement of force and power capacity (i.e. early acceleration), than more traditional, lighter loading protocols designed to develop the application of force at high velocities.

## Methods

### Participants and protocol

Participants were recruited, tested and trained at two locations: Finland and France. Both soccer [15 males; 27.1±4.8 years; 176±3.6 cm; mean±SD], and rugby [9 males and 12 females; 27.1±2.3; 175±9.7 cm; mean±SD] players volunteered to the study. They were all currently competing at club regional level (and within their national team for 5 rugby athletes) level and uninjured (<3 months pre-testing). All soccer players and all rugby players belonged to the same teams, and they performed the very same soccer/rugby program (training sessions and games) in addition to the sprint intervention. Soccer players did not perform any additional or gym-based strength work, and rugby players (both male and female) performed one gym-based strength maintenance session per week, with same content for all. Thus, any fluctuation would be included within the results of all athletes as they were split into the different analysis groups. The remaining team specific training was two soccer sessions (one rather intense and one of lower intensity) and one official game per week for the soccer players, and two to three rugby training sessions for the rugby players, with one game during the weekend. The intervention was performed in-season for both soccer and rugby players.

The procedures and methods used at each testing location were identical, with the exception of how horizontal resistance was applied to the athlete and running velocity sampled. Soccer players used a resisted sled while rugby players used a portable robotic resistance device (1080 Sprint, 1080 Motion, Lidingö, Sweden). All athletes were accustomed with the testing and training procedures and followed a progressive habituation training to heavy resistance prior to the beginning of the study. This study was based on the assessment, and subsequent prescription, of horizontally applied resistance based on the operational procedures outlined in recent research [14]. The athletes were individually assessed for horizontal force-velocity and load-velocity profiles, by utilizing a battery of sprints against increasing resistance (sled loads or robotic resistance). Rigorous pilot testing was performed before deciding on the resistance modalities. Both resistive techniques were able to provide targeted and constant decrements in maximal resisted velocity–the likes of which is the essential target of applying loading parameters, whatever the modality–and consequently presented very similar load/resistance-velocity linear relationships. Since the methodology of this study was based on the sprinting kinetics developed at maximum resisted velocity [14, 16], we posit that the modality of resistance had a negligible effect, provided that the resistance experienced by the athlete was constant and measureable at peak running velocity.

Athletes were divided randomly into training groups, within each team (soccer, male rugby and female rugby), so that equal numbers of players formed the two intervention groups. This random assignment ensured similar base values of maximal power output, gender and sport practice. Moreover, this allocation procedure ensured that players in both groups followed similar additional training content (soccer or rugby training, gym-based strength training or official games) than their teammates from the other group. Because of the equal separation of athletes to training groups, any differences in activities due to athlete code (e.g. increased weight training volume in rugby player) should be accounted for in the group allocation and subsequent results. The training intervention comprised of a 12-session training intervention at a loading protocol that either represented a 10% decrement in their individual maximum velocity (*L*_10_; *n* = 18), or at their individual optimal loading for maximal power (*L*_opt_; *n* = 18). Ethical approval was provided by the University of Jyväskylä Ethical Committee, and was performed in accordance with the Declaration of Helsinki. Written informed consent was obtained from the subjects prior to the study.

### Equipment

#### Soccer players

To provide resistance, athletes were harnessed at their waist and shoulders (attachment point mid-low back via 3-m non-elastic tether) to a heavy-duty, custom-made sprint sled loaded with a selection of weight plates. Sprinting performance was measured by a sports radar gun (Model: Stalker ATS II, Applied Concepts, Dallas, TX, USA), attached to a tripod set at 5 m and a height of 1 m, collecting outward bound velocity-time data at 46.9 Hz. Training equipment was identical to that used during testing, with the exception of the radar gun (which was only used during pre-, mid- and post-testing). The device has been widely used in the assessment of sprinting speed [24], including in resisted sprinting using loads equating that utilized in the current study [14].

#### Rugby players

The resistance used for the assessment, and subsequent training, of rugby players was applied using a robotic resistance device (1080 Sprint) featuring a servo motor (2000 RPM OMRON G5 Series Motor, OMORON Corporation, Kyoto, Japan) to provide resistance modes in 1 kg increments (1–30 kg load range). The motor is attached to a composite fiber cord that is wrapped around a spool, and attached to the athlete by a hip harness around the pelvis. During operation, the device was placed on the ground, secured by a selection of weight plates so as to render it immovable. The isotonic resistance mode was used and horizontal resistance was selected in 1 kg increments closest to those determined by the testing (%BM) and training protocol (*L*_opt_ and *L*_10_). Instantaneous velocity time data were collected from the manufacturer software at a rate of 333-Hz. This device has been used in a recently published resisted sprint study [25].

### Pre- and post-training testing procedures

All testing procedures were completed on grass fields, where the athletes subsequently performed their training. Athletes wore footwear typical to a maximal sprinting session. A standardised ~30-min warm-up including jogging, dynamic stretching, and submaximal 45-m stride outs (70, 80 and 90% of maximal self-selected effort) was performed. A 5-min active-recovery period directly preceded the commencement of testing, during which procedures were verbally recommunicated. The testing battery consisted of two ‘unresisted’ maximal 30-m sprints, and four sprints performed with increasing resistive loads up to maximal achievable velocity (*v*_max_) with a 5-min passive rest between trials.

Each trial required the athlete to take up a standing split-stance, behind a marked line, and sprint forward without any pre-start backward movement. When performing a resisted trial, athletes were instructed to ‘lean-in’ to the tether, and take up all slack before any initial forward movement, to ensure there was no ‘jerking’ or ‘bouncing’ of the sled. Athletes were instructed to be as ‘forward’ as possible, to eliminate any backward movement or countermovement that might affect their sprinting results. Verbal encouragement was provided to ensure a full maximal-effort throughout each trial.

#### Loading selection, and sprint distance

Five sprinting conditions were prescribed for each athlete: unresisted, 25, 50, 75 and 100% BM. Absolute loading parameters were used in the sled condition, and converted to relative resistance (0.35 conversion coefficient, provided by the manufacturer) in the robotic resistance condition. For example, if an athlete required a 40-kg load (50% of BM), the resistance programmed into the machine was set to 14-kg. The span of loading parameters was selected to provide a wide array of data for each athlete and to enable the accurate plotting of load-velocity relationships. Distances for each load were modeled based on previous research [14] and pilot testing of what was required to reach maximal velocity in the participants tested, as follows: 30-m unresisted, 30-m at 25%; 20-m at 50%; 20-m at 75%; 15-m at 100% BM or its´ 1080 Sprint equivalents.

### Data analysis

**Fig 2:**
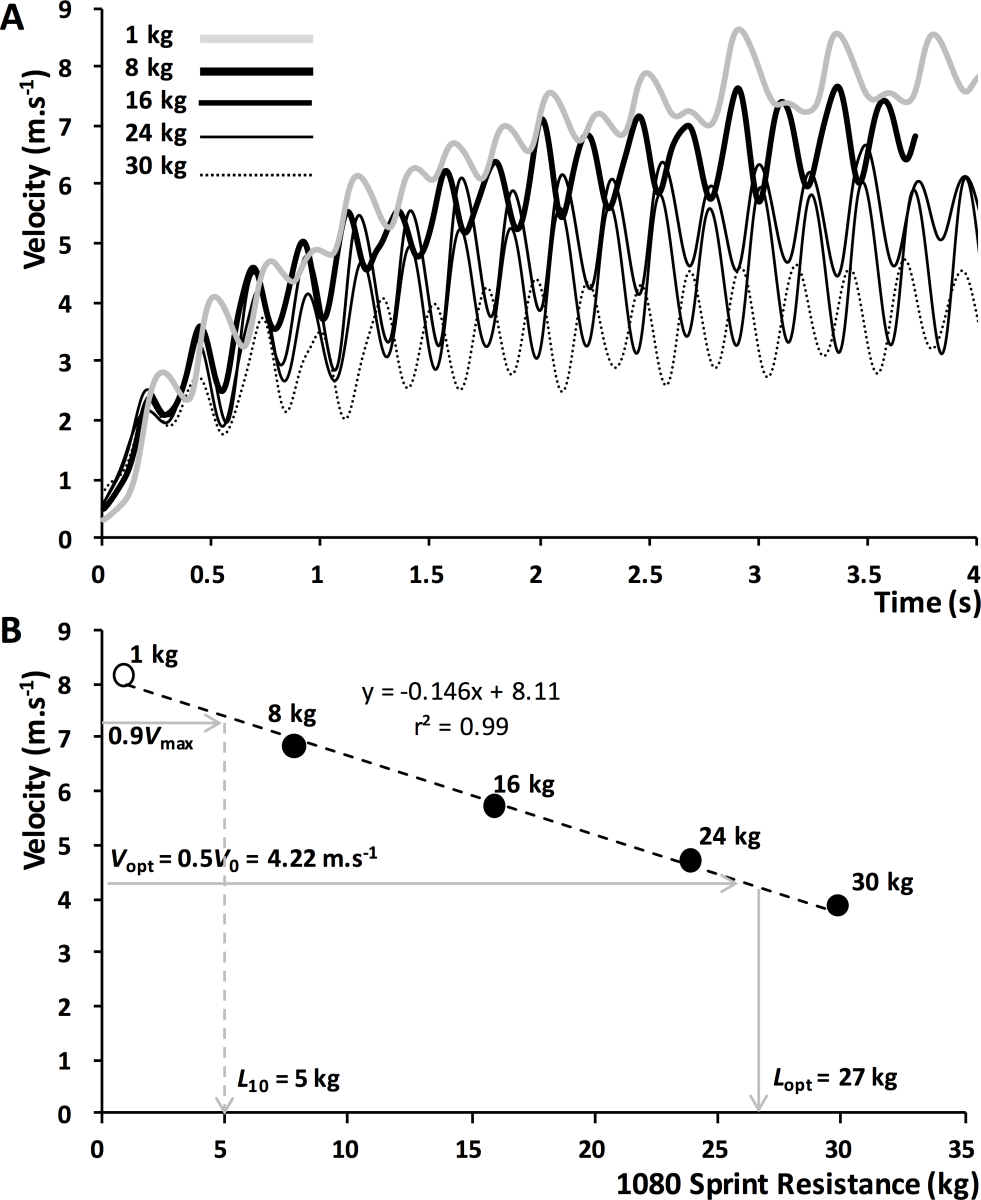
**A.** Running velocity measured with the 1080 Sprint device during resisted sprint acceleration, against loads corresponding to unresisted (minimal load of 1-kg), and 25, 50, 75 and 100% BM in a 1.73 m, 95-kg rugby player. **B.** maximal velocity was averaged for the last 2 s of each sprint and plotted against load to obtain the linear load-velocity profile, from which optimal load (*L*_opt_) and the load that induced a 10% decrease in maximal velocity (*L*_10_) were computed. Note that *L*_opt_ is produced at optimal velocity *v*_opt_ = 0.5*v*_0_ [14], data described in **Fig 1**.

Two types of data processing were applied in this study: (i) the assessment of sprint mechanical outputs from the acceleration phase of an unresisted sprint (see details below and in Fig 1C); and (ii) computation of individual loading parameters from multiple resisted sprints combined into a load-velocity relationship (Fig 2B).

#### Sprint mechanical outputs

For each unresisted trial, external horizontal force production was modelled from centre of mass movement using a validated method (described in detail elsewhere [13]). Briefly, a mono-exponential function was applied to raw velocity-time data [7] using a purpose-built software platform (Build version: 14.0, National Instruments Corp., Austin, TX, USA). From this point, macroscopically the acceleration of the athlete’s centre-of-mass can be calculated with respect to changing *v*_h_ over time. Net horizontal antero-posterior ground reaction forces (*F*_h_) can be modelled by considering the total system mass (*m*; in this case, the body-mass of each given athlete), and aerodynamic friction force (*F*_aero_) [7]:

$$F_{h}=m\cdot a+F_{aero}$$ (1)

Horizontal power (*P*_h_) is then modelled as the product of *F*_h_ and *v*_h_:

$$P_{h}=F_{h}\cdot v_{h}$$ (2)

From this point, Fv and Pv relationships were generated by fitting *F*_h_ and *v*_h_ data with least-squares linear regressions, and *P*_h_ and *v*_h_ data with 2^nd^ order polynomial fits. *F*_0_, *v*_0_ and *S*_Fv_ were determined as the *x* and *y* intercepts of the linear regression and the slope (respectively). *P*_max_ was determined as the optimal combination between *F*_0_ and *v*_0_ ([*F*_0_, *v*_0_]/4) [7]. The optimal conditions for power (*v*_opt_ and *F*_opt_) were calculated at the point of *P*_max_, corresponding to 0.5·*v*_0_ and 0.5·*F*_0_. A decrement of 10% from *v*_max_ was also calculated for application to the load-velocity profile, which will be discussed in the following section. Ground reaction force vector orientation (elsewhere termed “mechanical effectiveness of ground force application” [26]) was quantified through the ratio of force (*RF*) [26], calculated as the ratio of the horizontally-oriented (antero-posterior) component (i.e. *F*_h_) to the total estimated ground-reaction force signal. Note that *RF* is expressed in percent, but is the mathematical equivalent of the angle of ground reaction force vector orientation. A high *RF* is equal to a more horizontally directed ground reaction force vector [26], and vice versa. The linear decrease in *RF* with velocity was calculated and presented as an index of ground reaction force vector orientation throughout the acceleration phase (*D*_RF_) [26].

#### Load-velocity, *L*_opt_ and *L*_10_ computations

The force-velocity relationship using multiple trials of resistive loads has been previously validated shown to be linear [14]. Consequently, an associated linear load-velocity profile can be created to represent a span of loading under which mechanical conditions can be targeted.

Peak averaged 2-s velocity (i.e. the average velocity attained and maintained for 2-s at maximum effort) were taken for each sled load the athlete sprinted against, and matched with the exact resistance protocol from each respective sprint (as close as possible to 25, 50, 75, and 100% of athlete’s average BM). The data were then fit with a least-square linear regression to generate an individualised load-velocity profile for each athlete (Fig 2). In this data, the unresisted sprint was included as either a ‘zero load’ condition, or against the minimal possible resistance provided by the equipment (for sleds and robotic resistance, respectively). These data were then combined with that calculated using the simple method [13], represented in Fig 1, to calculate individualised training parameters to be applied for each training group. In the case of the experimental group training at optimal loading (*L*_opt_), the velocity at which *P*_max_ was produced (*v*_opt_) was substituted into the equation determined from the linear regression to provide the loading that corresponded to this velocity. The same method was applied to the group training at a 10% decreased velocity, where the loading parameter corresponding to a 10% decrement from the unresisted sprint maximum velocity (*v*_max_) was determined by applying entering this parameter into the linear regression. Specifically, the loading parameters for the experimental group were calculated based on the fact that (i) the corresponding running velocity is *v*_opt_ = 0.5*v*_0_ and (ii) the linear load-*v*_max_ relationship allows calculation of *L*_opt_ from the known *v*_opt_. Using the same approach, for the control group, *L*_10_ was determined using the same load-*v*_max_ relationship. In this case, *L*_10_ was determined as the loading corresponding to 0.9*·v*_max_. As a result, each athlete was provided with individualised resisted sprint training parameters that constituted a specific mechanical condition experienced during an unresisted sprint.

### Application of training loads, and training modalities

Due to a lack of information on the best method of applying resisted sprint overload, the training protocol was the same as presented in a recent pilot study in soccer players [22].

After two sessions of familiarization to heavy and very heavy loads over the span of two weeks, athletes were subject to a 12-session block of testing and training (Table 1). During sessions 1 and 12, the optimal load (*L*_opt_) and the load inducing a 10% decrease in maximal speed (*L*_10_) were determined for each player as described in Figs 1 and 2. Sessions 2 to 10 consisted of training (10 repetitions of 20 m resisted sprints, separated by approximately 5 min passive rest). At sessions 1, 6 (mid-program) and 12, the sprint force-velocity-power profiles were determined using the field method of [13] over 2 unresisted 30-m sprints.

**Table 1:** Study timeline.

Session/Week	Force-velocity-power profile assessment	Control Group	Experimental Group	
		*L*_10_ × 20-m	*L*_10_ × 20-m	*L*_opt_ × 20-m
1	2 unresisted × 30-m 4 loaded sprints (25, 50, 75, 100% BM)	-	-	-
2	-	10	8	2
3	-	10	6	4
4	-	10	4	6
5	-	10	2	8
6	2 unresisted × 30-m	10	0	10
7	-	10	0	10
8	-	10	0	10
9	-	10	0	10
10	-	10	0	10
11	-	-	-	-
12	Same testing protocol as session/week 1	-	-	-

BM: body mass; *L*_10_: light load used by the control group; *L*_opt_: optimal load used by the experimental group

### Statistical analysis

Descriptive statistics are presented as means ± standard deviation. Changes in athlete scores were evaluated using effect sizes (ES) and 90% confidence limits. Magnitude-based inferences were calculated using modified statistical Excel spreadsheets from sportsci.org (*xPostOnlyCrossover*.*xls*; *xParallelGroupsTrial*.*xls*). Two separate statistical methods were used to assess the effectiveness of each method of training. Pre- post-analysis was performed on each group’s data, to provide a clear effect of whether there were substantial and clear changes as a result of the training intervention. A second parallel group trials assessment compared the interventions. The thresholds used to interpret the magnitude of effects measured were based on the work of Hopkins and colleagues (sportsci.org) [27]. Probabilities that differences were higher, lower or similar to the smallest worthwhile difference (preset at a value of 0.20) were evaluated qualitatively as: possibly, 25–74.9%; likely, 75–94.9%, very likely, 95–99.5%; most (extremely) likely, >99.5%. The true difference was assessed as unclear if the chance of both higher and lower values was >5%.

## Results

All Fv relationships, pre- and post-training were well fitted by linear regressions (all *r*^2^>0.98, typical example in Fig 1C). Similar results were obtained for all linear load-velocity relationships for both the radar and the 1080 Sprint protocols (all *r*^2^>0.924).

The main result from the group comparison (*L*_opt_ versus *L*_10_ between-group differences) showed a likely trivial between-group difference in 20-m performance and *P*_max_ increase post-training. All results are presented in Table 2, with between-groups differences presented in Table 3.

**Table 2:** Athlete body-mass, mechanical, technical and performance sprint variables during pre- and post-testing for the *L*_10_ and *L*_opt_ groups.

	*L*_10_ (*n* = 18)					*L*_opt_ group (*n* = 18)				
	Pre	Post	Post–Pre			Pre	Post	Post–Pre		
	$\bar{x}$ ± SD	$\bar{x}$ ± SD	%Δ ± SD	*ES; ±90% CL*	*Inference*	$\bar{x}$ ± SD	$\bar{x}$ ± SD	%Δ ± SD	*ES; ±90% CL*	*Inference*
Body-mass (kg)	76.5 ± 14.6	76.8 ± 15.2	0.32 ± 1.50	*0*.*02; ±0*.*03*	Trivial**** *(neutral)*	81.9 ± 17.1	82.1 ± 16.2	0.43 ± 1.57	*0*.*01; ±0*.*03*	Trivial**** *(neutral)*
*v*_0_ (m·s^-1^)	7.86 ± 0.90	7.95 ± 0.88	1.19 ± 2.83	*0*.*09; ±0*.*10*	Trivial*** *(neutral)*	7.93 ± 0.86	8.16 ± 0.91	2.96 ± 2.90	*0*.*26; ±0*.*11*	Small** *(positive)*
*F*_0_ (N·kg^-1^)	6.75 ± 1.07	7.10 ± 0.88	6.49 ± 12.99	*0*.*32; ±0*.*32*	Small* *(positive)*	6.77 ± 1.00	6.90 ± 0.90	2.78 ± 10.23	*0*.*13; ±0*.*29*	Trivial* *(neutral)*
*P*_max_ (W·kg^-1^)	13.3 ± 3.2	14.2 ± 3.0	7.48 ± 11.90	*0*.*25; ±0*.*20*	Small* *(positive)*	13.5 ± 3.2	14.1 ± 3.0	5.58 ± 9.54	*0*.*17; ±0*.*17*	Trivial* *(neutral)*
S_*Fv*_ (%)	-65.5 ± 13.0	-68.6 ± 14.4	5.73 ± 14.07	*-0*.*23; ±0*.*30*	Small*(positive)	-69.9 ± 15.7	-69.4 ± 14.1	0.51 ± 12.19	*0*.*03; ±0*.*21*	Trivial* *(neutral)*
*RF*_max_ (%)	42.5 ± 7.1	46.9 ± 5.1	12.15 ± 15.50	*0*.*59; ±0*.*27*	Small***(positive)	43.7 ± 7.4	46.6 ± 5.7	7.96 ± 12.52	*0*.*37; ±0*.*26*	Small** *(positive)*
*D*_RF_	-8.1 ± 1.1	-8.3 ± 0.8	3.97 ± 14.50	*-0*.*17; ±0*.*39*	***Unclear***	-7.9 ± 0.6	-7.8 ± 0.8	-1.04 ± 10.99	*0*.*18; ±0*.*59*	***Unclear***
5-m (s)	1.50 ± 0.13	1.46 ± 0.11	-2.28 ± 4.94	*-0*.*28; ±0*.*22*	Small* *(positive)*	1.49 ± 0.14	1.47 ± 0.11	-1.40 ± 4.11	*-0*.*17; ±0*.*17*	Trivial* *(neutral)*
10-m (s)	2.32 ± 0.20	2.27 ± 0.18	-2.11 ± 4.33	*-0*.*24; ±0*.*19*	Small* *(positive)*	2.31 ± 0.22	2.27 ± 0.18	-1.46 ± 3.60	*-0*.*16; ±0*.*14*	Trivial* *(neutral)*
20-m (s)	3.77 ± 0.35	3.70 ± 0.33	-1.96 ± 3.31	*-0*.*21; ±0*.*13*	Small* *(positive)*	3.75 ± 0.38	3.67 ± 0.33	-1.81 ± 2.78	*-0*.*18; ±0*.*11*	Trivial* *(neutral)*
*v*_max_ (m·s^-1^)	7.40 ± 0.83	7.53 ± 0.81	1.78 ± 2.05	*0*.*15; ±0*.*07*	Trivial** *(positive)*	7.48 ± 0.84	7.70 ± 0.83	2.99 ± 2.31	*0*.*25; ±0*.*08*	Small** *(positive)*

Values are mean ± standard deviation, percent change ± standard deviation and standardized effect size; ±90% confidence limits. Abbreviations: *n*, sample size; $\bar{x}$, mean; SD, standard deviation, %Δ, percent change; ES, effect size; 90% CL, 90% confidence limits; kg, kilogram; *v*_0_, maximal theoretical running velocity; m, meter; s, second; *F*_0_, maximal theoretical horizontal force; N, newton; *P*_max_, maximal power; W, watt; S_*Fv*_, Slope of the linear force-velocity relationship; *RF*_max_, maximal ratio of force; *D*_RF_, decrease in the ratio of force; *v*_max_, maximal running velocity. Qualitative inferences are trivial (< 0.20), small (0.20 –< 0.60) and moderate (0.60 –< 1.20)

* possibly, 25 –< 75

** likely, 75 –< 95%

*** very likely, 95 –< 99.5%

**** most likely, > 99.5%. Positive, neutral and negative descriptors qualitatively describe the change between post- and pre-values and its importance relative to the specific variable.

**Table 3:** Post–pre changes in athlete body-mass, mechanical, technical and performance sprint variables between the *L*_10_ and *L*_opt_ groups.

	Post–Pre group change		*L*_opt_ group–*L*_10_ group	
	*L*_10_ group (*n* = 18)	*L*_opt_ group (*n* = 18)	*ES; ±90% CL*	*Inference*
	$\bar{x}$ ± SD	$\bar{x}$ ± SD		
Body-mass (kg)	0.32 ± 1.28	0.18 ± 1.36	*-0*.*01; ±0*.*05*	Trivial**** *(neutral)*
*v*_0_ (m·s^-1^)	0.09 ± 0.22	0.23 ± 0.23	*0*.*16; ±0*.*14*	Trivial* *(neutral)*
*F*_0_ (N·kg^-1^)	0.35 ± 0.88	0.13 ± 0.75	*-0*.*21; ±0*.*44*	***Unclear***
*P*_max_ (W·kg^-1^)	0.84 ± 1.64	0.59 ± 1.40	*-0*.*08; ±0*.*27*	Trivial** *(neutral)*
S_*Fv*_ (%)	-3.16 ± 10.11	0.43 ± 8.24	*0*.*24; ±0*.*35*	Small* *(positive)*
*RF*_max_ (%)	4.36 ± 4.95	2.87 ± 4.82	*-0*.*20; ±0*.*38*	Small* *(positive)*
*D*_RF_	-0.20 ± 1.12	-0.11 ± 0.86	*0*.*35; ±0*.*63*	***Unclear***
5-m (s)	-0.037 ± 0.072	-0.024 ± 0.059	*0*.*10; ±0*.*28*	Trivial* *(neutral)*
10-m (s)	-0.053 ± 0.098	-0.038 ± 0.081	*0*.*07; ±0*.*24*	Trivial** *(neutral)*
20-m (s)	-0.078 ± 0.122	-0.073 ± 0.105	*0*.*01; ±0*.*17*	Trivial** *(neutral)*
*v*_max_ (m·s^-1^)	0.13 ± 0.15	0.22 ± 0.16	*0*.*11; ±0*.*10*	Trivial** *(neutral)*

Values are mean ± standard deviation and standardized effect size; ±90% confidence limits. Abbreviations: *n*, sample size; $\bar{x}$, mean; SD, standard deviation, ES, effect size; 90% CL, 90% confidence limits; kg, kilogram; *v*_0_, maximal theoretical running velocity; m, meter; s, second; *F*_0_, maximal theoretical horizontal force; N, newton; *P*_max_, maximal power; W, watt; S_*Fv*_, Slope of the linear force-velocity relationship; *RF*_max_, maximal ratio of force; *D*_RF_, decrease in the ratio of force; *v*_max_, maximal running velocity. Qualitative inferences are trivial (< 0.20), small (0.20 –< 0.60) and moderate (0.60 –< 1.20):

* possibly, 25 –< 75

** likely, 75 –< 95

*** very likely, 95 –< 99.5%

**** most likely, > 99.5%. Positive and neutral descriptors qualitatively describe the change between the post–pre changes for the *L*_opt_ and *L*_10_ group values and its importance relative to the specific variable.

## Discussion

Overall, both optimally loaded (*L*_opt_) and ‘10% decrease in *v*_max_’ loaded interventions (*L*_10_) provided beneficial effects in a range of performance variables (Table 2). The magnitude of changes experienced were often marginal (small and trivial), and there was a lack of clear distinction in the group effects between the training cohorts (Table 3). Our initial hypothesis was that conditions of high horizontal force exhibited during training would transfer into a linked increase in the corresponding mechanical capacity (i.e. increased *F*_0_), with training in conditions of lower force resulting in emphasised improvements in the opposite capacity (*v*_0_). Instead, what we observed were clear trends for small changes in both force and velocity capabilities as a result of both modalities of training. Among the possible explanations of this unexpected result is a possible interaction effect between pre-test values of Fv profiles, and the timing of post-training measurements, that was common to all subjects; this and other points will be discussed towards the end of this article.

A detailed commentary of the methods, including suggestions of how they may be best applied in a practical context, is available as supplementary material (S1 Table and S1 Text). While this section includes discussion of the results as they pertain to the outcome of the training implementation, we believe this additional content is important for practitioners wishing to implement the methods in the field, without an ability to access the types of equipment widely available to researchers.

*L*_10_ produced small measureable increases in practical performance measures, with a reduction in sprint times between -2.28 to -1.96%. However, *L*_opt_ produced negligible group changes overall -1.40 to -1.81%. The changes observed were within the range (-0.5 to -9.12%) reported in a recent systematic review [18] from a range of resisted sprint training interventions. The comparison between the effects of the training interventions were largely inconclusive, with the majority of comparative analyses requiring more data to provide clear statistical inferences (changes are unclear; Table 3). These results appear to stand in opposition to the theory that training in conditions that represent a particular mechanical condition will transfer to changes in said condition [8, 15]. Indeed, a recent example in jumping [12] clearly showed that training in the conditions of high force resulted in developments of this capacity. Moreover, previous literature on resisted sprinting (albeit limited in nature) remains unclear, and more research is required to test whether heavier training may specifically benefit the development of acceleration based stimuli [18]. As such, these somewhat counterintuitive results will be discussed further in the paragraphs following.

The result of lighter loading providing a platform for the development of maximum force, and conversely heavier loading developing velocity, appears to refute our initial hypothesis of specific mechanical adaptations in sprint training. There are some factors that we believe may help clarify these unexpected results. Firstly, the change scores and associated inferences were relatively similar (although clear) between training protocols (Table 2). While the statistical interpretation for some effects was different (i.e. trivial vs. small), it could be questioned whether these results were separated by enough of a margin to be practically worthwhile. Regardless, this simply would appear to support a conclusion that loading may not substantially affect the outcomes of this manner of resistive sprint training. One possible explanation for this unexpected outcome may be found in the spread of results observed in the team sport players studied. For example, while the effects of the training interventions were different in respect to their effects on *RF*_%max_ (small inference, likely negative), the range of change scores observed from the groups were wide (12.15 ± 15.50% and 7.96 ± 12.52%, for *L*_10_ and *L*_opt_ changes, respectively). These results may suggest that while there were no statistically measureable differences in the groups at baseline with regards to practical performance measures, the adaptations experienced by the athletes studied were highly individualised.

We theorise a scenario where athletes adapted to the stimuli provided to them based on the level and balance of Fv capacities exhibited pre-test (i.e. their initial Fv profile). For example, athletes with a more velocity-oriented profile (i.e. less negative *S*_Fv_ or flatter linear Fv relationship) may have improved their force capacity, regardless of the magnitude of resistive stimulus applied; it is important to note that resisted sprint training using loading constituting *L*_10_ training (or indeed lighter) may represent force-dominant training for some athlete presenting a low *F*_0_/*RF*_%max_ profile. The unfortunate factor about this theory, is that due to the limited information on sprint Fv profile it is difficult to qualify a priori individual strengths and weaknesses in mechanical capacities. One possible avenue of such an approach is comparing individual scores to a group average/median value, however due to a current lack of normative data these types of analyses are not possible. In jumping the existence of an individualized optimal balance between mechanical capacities has been proven [2], and it is possible that a model of individual optimal Fv capacities applies to that developed during a sprint running acceleration phase. If such an approach is validated in sprinting, an athlete’s particular orientation with regards to this theoretical optimal profile may influence their results from a given training program. This may, partially explain why some athletes in this study improved and not others, even when presented with a greater ‘force-oriented’ stimulus (*L*_opt_ sled). To further explore this theory, we tested the correlation between individual pre-score *S*_Fv_ and training-induced changes in *F*_0_ and observed a significant interaction effect (*r* = 0.39; *P*<0.02). Similarly, changes in *RF*_%max_ appeared to be related to the pre-intervention levels expressed in each individual (*r* = -0.66; P<0.0001). Simply, changes in *RF*_%max_ were greater for participants who presented lower values pre-intervention, whatever training method they followed. Consequently, it seems logical that an athlete expressing a low *RF*_%max_ value would improve his *RF*_%max_ following training using a lighter stimuli (*L*_10_) than an athlete with high *RF*_%max_ value following heavier training (*L*_opt_). However, no statistically observable differences were found between groups in these variables at baseline. Taken present and previous findings together, the quest for effective training load stimuli to improve sprint mechanical output and performance requires a more individualized approach as to the specific needs (and thus margins of improvement) of athletes. These theories require further investigation, and we recommend that in the future, athletes should be assigned to intervention group(s) based on their initial Fv characteristics [12], rather than randomly as in the current pilot study.

Another possible consideration is the different nature of what happened within sprints during the resisted sprint stimulus *before* the athletes reached maximal resisted speed (note: maximum resisted velocity is methodologically the point around which loading was profiled, allocated and trained). It is possible that while the conditions at maximum resisted velocity targeted specific adaptations in the way that we hypothesized [15], the time spent before that peak was attained (i.e. 2 to 4 seconds depending on the participants, Fig 2A) while *accelerating* confounded the results. That is, during each sprint of the training program, the *L*_opt_ group probably spent ~4 seconds below *v*_opt_ (thus training at *v*_opt_ only a very short amount of time in the sprint), and as a consequence potentially not enough time at *P*_max_. (~2–3 seconds). Perhaps if the athletes had performed less sprints, but more time at *v*_opt_ (~6 seconds) we may have seen different adaptations. There seems to be a trade-off between accelerating from zero to *v*_opt_ and maintaining *v*_opt_ at the targeted *P*_max_ velocity. Although it might be technically challenging to use such a setting in sprint running, future research could investigate the effect of a resistance onset applied only once the athletes have reached *v*_opt_ after an unresisted acceleration, as in sprint cycling [28, 29]. This type of investigation could clarify the role played by the acceleration phase work against *L*_opt_ compared to the work performed in the *F*_opt_ / *v*_opt_ conditions as a stimulus to improve *P*_max_.

Finally, all athletes were tested post-training at the same time after the last session of the protocol. It cannot be ruled out that the systematic 2-week time window used between the end-of-training and post-testing was not optimal for some athletes. It is possible that the adaptation necessary to transfer heavy-sled resistance training over several sessions to improve sprint acceleration performance and mechanical outputs may have taken longer in some athletes. As seen in elite rugby players following a development and tapering protocol [30], and confirmed by our experience with elite players using heavy-resistance sprint training (unpublished data), sprint performance and physical qualities show variable kinetics of ‘peak adaptation’. Such variability is included in a ‘single post-training measurement session’ design. However, since this is the case in most sprint training studies, further research should determine the training-induced adaptation kinetics after heavy-sled resisted training in order to verify if, and to what extent, athletes show peak adaptation windows and between-participants variability in the occurrence of these peaks. Overall, the efficacy of a range of periodization protocols on adaptations, including total sprinting load (per session, and macro-cycle length), and tapering lengths (i.e. time course of adaptations in following weeks), should be studied in more detail, to allow for peak performance following a resisted sprint training program.

### Limitations and future directions

There are a number of limitations that need to be considered in the interpretation of the results of this study. Namely, while the aims of this research were to provide a clear insight into the effects of training using differing loading protocols and clarify the effects of training at high/low loads, we believe the sample studied (e.g. size, sporting codes, mixed-sex) may have influenced our results. Future researchers should look to replicate similar studies with larger and more homogenous cohorts (e.g. 100-m sprinters). In addition, no group was used in this study with players performing an equivalent program of unresisted (no additional load) sprints. Thus we cannot compare the results obtained in the *L*_opt_ and *L*_10_ groups to those of standard, unresisted sprint training. This decision was made due to the constraints inherent to an in-season study in a team sport competition context, and because such a sprint training program resulted overall in trivial changes in sprint acceleration mechanical outputs and performance in the control group of a similar protocol [22]. Partly for the same reason (in-season intervention in competitors), the *L*_opt_ program (Table 1) did not include sprints at *L*_opt_ exclusively, but a load progression was set over the first four weeks of training. This was performed in addition to the familiarization sessions prior to the intervention to ensure a safe and progressive adaptation to the high-load stimulus. One consequence was that the *L*_opt_ group did a total of 80 sprints at *L*_opt_ and 20 sprints at *L*_10_ over the 10 weeks of training versus 100 sprints at *L*_10_ in the *L*_10_ group. Although we do not think this is a major bias, further intervention should use perfectly balanced training content between groups. While the surfaces used for training were controlled and maintained to the best of our ability, with weather relatively stable throughout the experiments, it is possible that minor variations in surface conditions may have affected the resistance experienced by the athletes using sled resistance [16]. Moreover, it is possible that the use of different methods of providing resistance to the athletes may have affected the results. While we assume that the two devices provided identical type resistance at their maximal resisted velocity, it is possible the actual characteristics of the resistance experienced by the athletes may have differed to a degree not captured by matched velocity measurement. To our knowledge, no research exists on the topic of differing methods of applying matched horizontal resistance, however the topic should be of interest to coaches looking at the best method applying such loading to meet their athletes’ specific needs.

## Conclusion

Seemingly in contrast to our hypothesis, the group effects of sprint training at optimal power did not appear to be substantially different than training using traditional lighter loading protocols. However, individual adaptations to the type of training imposed were varied, leading us to conclude that pre-training Fv profile (among other confounding factors) may have contributed to the results observed in the athletes. Despite seeming lack of clarity in these results, it should be noted that both resisted-sprint training protocols were likely to improve performance after a short training intervention in already sprint trained athletes. We hope the results of this study will add to the developing narrative of resisted sprint literature, and will serve to guide future, well-designed research in the area of individualised programming for resisted sprinting.

## Supporting information

S1 Table Spreadsheet for optimal loading computation. (XLSX)

S1 Text Complementary methodological considerations. (DOCX)
