# Supplementary material for: Training at maximal power in resisted sprinting: Optimal load determination methodology and pilot results in team sport athletes
Source: PLoS One. 2018 Apr 11;13(4):e0195477. doi: 10.1371/journal.pone.0195477 (PMC5895020; doi:10.1371/journal.pone.0195477)
Supplement: S1 Text — (DOCX) [file pone.0195477.s002.docx]

# Supplementary material

The following commentary will provide an overview of considerations for practitioners wishing to replicate the methods used in this study to assess and prescribed loaded based on each athlete’s individual Fv profile. In essence, the method follows that published in assessing force-velocity profiles from multiple trials of resisted sprints [[1](#_ENREF_1)], with the exception of considering friction to calculate horizontal force at peak velocity. While the method itself was originally applied to resisted sprinting using a sprinting sled, it can be applied to any resistance that is easily measureable and practically applicable. The method follows two distinct steps (illustrated in Figure 1 and 2): (i) generation of a load-velocity (Lv) profile from a range of unloaded and resisted sprints (under external load), and; (ii) application of a simple formula to extract optimal velocity, and apply this to the load-velocity relationship to calculate *L_opt_*. The two steps will be described in detail below, however there are several requirements which should be broached at the outset.

### Generation of the horizontal load-velocity profile

In essence, the generation of a horizontal load-velocity profile is simple, and not dissimilar to that widely published in the strength and conditioning literature (e.g. variants of velocity based training [[2](#_ENREF_2), [3](#_ENREF_3)]). Simply stated, the user must perform a selection of sprints up to maximum resisted velocity under a variety of resistive loading conditions. However, in order to profile an athletes horizontal load-velocity relationship the three main requirements are: (i) a reasonable friction constant surface to test and train on; (ii) controllable, easily applicable and friction constant resistance method, and; (iii) measurement technology capable of accurately capturing maximum resisted velocity. Depending on the modality through which resistance is applied, these factors will be more or less important. For example, using resisted sleds to generate a load velocity profile requires an innate knowledge of factor (i), however robotic resistance circumvents this factor due to the method through which resistance is applied and controlled. Regardless, these factors require consideration in the accurate and repeatable assessment of training load, and hence will be discussed in detail.

### Measurement technology

The method of assessing the multiple-trial load-velocity relationship is framed on the performance of the athlete, under load, expressed at their given resisted maximum velocity. The idea being that if we are able to select a given stimuli for this instance, we are able to target and extend the experience of these conditions simply by having the athlete build to and maintain this maximum resisted effort. This necessitates an accurate measure of the exact moment during which the athlete reaches this velocity, be it with or without resistance. The simplest means of acquiring this information is by using a technology that provides relatively instantaneous velocity-time data throughout a sprint phase, such as sports radar devices [[4](#_ENREF_4)], laser [[5](#_ENREF_5)], or systems utilizing cables attached to the sprinter [[6](#_ENREF_6)]. Using such devices simply requires the user to set up behind the athlete performing their sprints (without the need to customize setup based on sprinting distance), collect data and then select the maximum point on the velocity-time curve. These devices are not yet widely used, however, and many coaches will not have access to such technology. Consequently, it is possible to attain similar data using common sports training equipment, albeit requiring some creativity.

It may be possible to use other alternatives to capture and rank an athlete’s performance, such as timing gates, GPS [[7](#_ENREF_7)], the robotic resistance device used in this study, other alternatives (e.g. video or video applications [[8](#_ENREF_8)]). The simplest and most repeatable way of attaining said data is to capture a reading of average velocity across two samples that is as close as possible to the athlete’s maximum resisted effort. In the example of timing gates (photoelectric cells), the idea would simply be to set a pair of gates at a distance at which each tested athlete can reach and maintain maximum resisted velocity. For example, one may set a pair of gates at the beginning of a 20 m sprint, and then two more sets of gates 5 m apart where it is estimated the athlete has reached and will maintain their maximum resisted effort. If more gates are available, a greater number of ‘sampling windows’ could be created, and the user would simply have to select the window with the highest average velocity. Once the gates are set up, the athletes should start at different distances behind the sampling window, depending on the load under which they are sprinting. However, because athletes will reach their maximum resisted velocity at different distances under increasing loads, the complexity with this method is the need to change the sprinting distance to ensure you capture this *v*_max_ plateau ‘window’ without fatigue. This may be feasible for practitioners who are dealing with a limited number of athletes in a testing session, however the individualized nature of this assessment likely limits the feasibility for those working in team-sport scenarios. Separation of athletes into homogenous groups (e.g. rugby forwards, and rugby backs) may enable the user to create distinct testing setups that meet the majority of athletes demands, while minimizing the time spent in organizing such a testing session. As highlighted earlier, technology offering a continuous measurement of velocity (e.g. laser or cable device) might be favorable and provide the greatest ease of measurement for the user.

Note that while the use of accurate technologies is advisable, it may be the case that any error that comes a product of either of these methods does not actually result in a worthwhile difference in adaptations to the athlete.

### Selection of loading magnitude, trial numbers, and trial distance

The aim of load selection and trial numbers is to provide an accurate and linear Lv profile, while working within the constraints of allocated testing time and resources. While individualized loading for profiling was used in the first iteration of these methods [[1](#_ENREF_1)], due to the proven linearity of the subsequent Lv data, it is not necessary to use individualized loading (e.g. percent of BM) parameters for assessment in homogenous cohorts. The magnitude of loading parameters should potentially be fairly disparate, and (if possible) exceed that which produces a 50% decrement in maximum resisted velocity to capture the peak of the power-velocity relationship [[1](#_ENREF_1)]. The exact loading will vary substantially depending on the surface used, however approximately 80% of body-mass, or the equivalent from robotic resistance, may provide an approximation to begin with [[1](#_ENREF_1), [9](#_ENREF_9)]. It is theoretically possible to provide an accurate Lv profile without utilizing these magnitudes of loads (particularly given the linearity *R*^2^>0.987 of the Fv relationship in resisted sprinting, albeit from 6 to 7 trials), however several factors will affect the success of this factor. Namely, the fewer trials used, the greater likelihood that error will contribute to the resulting Lv profile. This may be particularly important to consider when profiling individuals who are not accustomed to the testing modality. Consequently, greater trials numbers will provide a more accurate profile, and therefore the most trials that can be feasibly measured without fatigue is suggested. Approximately 4 disparate loading conditions is recommended to provide a clear linear Lv profile [[10](#_ENREF_10)], however there is a possibility that much fewer loads may suffice [[11](#_ENREF_11)]. We recommend using an unloaded sprint as one of the testing conditions, or a very-light loading protocol, to provide an accurate point close to the unloaded condition for later analyses. Obviously, athletes must be familiar with heavy and very-heavy sled resistance sprinting to ensure data reliability and safe testing and training.

The distance required for each sprint will vary greatly, depending on the loading parameter applied. The key is to not make athletes sprint too far unnecessarily, while concurrently not potentially missing the moment at which the athlete reaches peak resisted velocity. Note that in the case of using technology that does not provide high sample velocity-time data, a distance of velocity maintenance (and hence an extended assessment window) may be necessary. Approximate guidelines have been published [[1](#_ENREF_1)] for track and field athletes (45 m unloaded, 40 m at 20%; 30 m at 40%; 30 m at 60%; 30 m at 80%; 20 m at 100%; and 20 m at 120% BM), and may be used as a starting point to manipulate.

### Selection of training parameters applicable to mechanical conditions experienced within an unloaded sprint trial (i.e. *P*_max_)

This step of the method combines the data compiled from the multiple resisted sprints, with orientational data calculated from the data measured, or estimated, in an unloaded sprinting condition. The idea is to take a measure of maximum theoretical velocity, from this determine the velocity at which maximum power was produced (i.e. 0.5·*v*_0_, or *v*_opt_), apply this variable to the linear Lv relationship to determine the loading corresponding with this velocity (*L*_opt_). The user can then train at loads greater or lesser than *L*_opt_ to hypothetically target various components of the Fv spectrum. In the same manner, you may select any load corresponding to a particular sprint phase (i.e. 6 m/s corresponding to mid-to-late acceleration phase in most athletes), and apply this to determine the individualized training parameters.

The user has three main options to ascertain the necessary data: the user may 1) select the ‘theoretical’ maximum velocity determined from the intercept of the load-velocity relationship; 2) use *v*_max_ determined from an unloaded sprint, or; 3) use the ‘true’ *v*_0_ value calculated using validated equations [[12](#_ENREF_12)] applied to an unloaded sprint. The former two methods represent a simpler option than the latter, but may include error. However, as noted earlier in this section, the degree to which this error is practically significant is yet to be clarified.

Notably, any training parameters determined using devices that generate resistance based on their interaction with the environment (i.e. sleds) are only specific to those exact conditions. Consequently, there is a need to be re-profiled in the case of training on different surfaces. Some methods somewhat circumvent this problem (i.e. robotic resistance), and these factors will be discussed in the following section.

### Note regarding friction and modes of resistance

When using traditional methods of resistance application for profiling horizontal force-velocity and load-velocity capacities for your athletes, you are at the mercy of friction. Because friction factors (e.g. size sled, material, surface type) can largely effect the magnitude and behaviour of the resistance experienced by the athlete, it is crucial to consider this in the assessment of horizontal training resistance. Namely, the use of the ‘friction’ based resistance dictates that the friction must be maintained across all testing and training occasions. Simply, the equipment and surface under which loading parameters are assessed must remain constant (or as much as possible) to that to be trained under. Training load magnitude must be considered, however, as a fluctuation in a friction coefficient between 0.4 and 0.5 [[13](#_ENREF_13)] may not be practically worthwhile to correct when sprinting with load constituting 10% of body-mass, but may present a problem when using loading protocols of greater magnitudes.

While sleds are arguably the most common and popular method of resistance for sprinting, there are an increasing number of alternatives that offer distinct advantages and disadvantages [[14](#_ENREF_14)]. For example, modular cable and rope pulley resistance systems enable the user to alter the resistance experienced by the athlete by applying a mechanical brake to the rope (e.g. Exer-Genie). While the rope may still be subject to changes in resistance due to moisture, or wear on the equipment, portable alternatives may provide an attractive proposition to those coaches who regularly train their athletes on different surfaces. There are now several robotic resistance devices (such as the example used in the current study) widely available. While much more costly than other more basic alternatives, these machines offer direct control of load (assisted and resisted) and measurement of many factors experienced by the athlete, including velocity, acceleration and force. Aside from not being subject to the same environmental fluctuations that plague traditional methods of resisted sprint training, the options afforded by these units provide invaluable control for athletes and coaches, including instantaneous feedback and analysis options which provides the types of analysis and prescription piloted in this (and previous) studies. This is a distinct advantage, and somewhat accounts for the large discrepancy in cost between methods – there is little need to purchase other speed testing equipment that is necessary to generate athlete profiles in the other methods. With that said, the only requirements for the method of load-velocity profiling are a controllable and practical device to provide resistance.

While this may exclude devices such as parachutes, many other alternatives may fit within these requirements. Further study is needed to clarify the actual effects of each on training outcomes.

### References:

1. Cross MR, Brughelli M, Samozino P, Brown SR, Morin JB. Optimal loading for maximising power during sled-resisted sprinting. Int J Sports Physiol Perform. 2017:1-25. doi: 10.1123/ijspp.2016-0362. PubMed PMID: 28051333.

2. Conceicao F, Fernandes J, Lewis M, Gonzalez-Badillo JJ, Jimenez-Reyes P. Movement velocity as a measure of exercise intensity in three lower limb exercises. J Sports Sci. 2016;34(12):1099-106. doi: 10.1080/02640414.2015.1090010. PubMed PMID: 26395837.

3. Gonzalez-Badillo JJ, Sanchez-Medina L. Movement velocity as a measure of loading intensity in resistance training. Int J Sports Med. 2010;31(5):347-52. doi: 10.1055/s-0030-1248333. PubMed PMID: 20180176.

4. Cross MR, Brughelli M, Brown SR, Samozino P, Gill ND, Cronin JB, et al. Mechanical properties of sprinting in elite rugby union and rugby league. Int J Sports Physiol Perform [Internet]. 2015 Sep; 10(6):[695-702 pp.].

5. Buchheit M, Samozino P, Glynn JA, Michael BS, Al Haddad H, Mendez-Villanueva A, et al. Mechanical determinants of acceleration and maximal sprinting speed in highly trained young soccer players. Journal of sports sciences. 2014;32(20):1906-13. doi: 10.1080/02640414.2014.965191.

6. Harris NK, Cronin J, Taylor K-L, Boris J, Sheppard JM. Understanding position transducer technology for strength and conditioning practitioners. Strength & Conditioning Journal. 2010;32(4):66-79.

7. Nagahara R, Botter A, Rejc E, Koido M, Shimizu T, Samozino P, et al. Concurrent validity of GPS for deriving mechanical properties of sprint acceleration. Int J Sports Physiol Perform. 2016;Published ahead of print. Epub 2016/03/24. doi: 10.1123/ijspp.2015-0566. PubMed PMID: 27002693.

8. Romero-Franco N, Jimenez-Reyes P, Castano-Zambudio A, Capelo-Ramirez F, Rodriguez-Juan JJ, Gonzalez-Hernandez J, et al. Sprint performance and mechanical outputs computed with an iPhone app: Comparison with existing reference methods. Eur J Sport Sci. 2017;17(4):386-92. doi: 10.1080/17461391.2016.1249031. PubMed PMID: 27806673.

9. Morin JB, Petrakos G, Jimenez-Reyes P, Brown SR, Samozino P, Cross MR. Very-heavy sled training for improving horizontal force output in soccer players. Int J Sports Physiol Perform. 2017;12(6):840-4. doi: 10.1123/ijspp.2016-0444. PubMed PMID: 27834560.

10. Morin JB, Samozino P. Interpreting power-force-velocity profiles for individualized and specific training. Int J Sports Physiol Perform. 2016;11(2):267-72. Epub 2015/12/24. doi: 10.1123/ijspp.2015-0638. PubMed PMID: 26694658.

11. Jaric S. Two-load method for distinguishing between muscle force, velocity, and power-producing capacities. Sports Medicine. 2016:1-5. doi: 10.1007/s40279-016-0531-z.

12. Samozino P, Rabita G, Dorel S, Slawinski J, Peyrot N, Saez de Villarreal E, et al. A simple method for measuring power, force, velocity properties, and mechanical effectiveness in sprint running. Scand J Med Sci Sports. 2016;26(6):648-58. doi: 10.1111/sms.12490. PubMed PMID: 25996964.

13. Cross MR, Tinwala F, Lenetsky S, Samozino P, Brughelli M, Morin JB. Determining friction and effective loading for sled sprinting. J Biomech. 2017;35(22):2198-203. doi: 10.1080/02640414.2016.1261178.

14. Cronin J, Hansen KT. Resisted Sprint Training for the Acceleration Phase of Sprinting. Strength Cond J. 2006;28(4):42-51. doi: 10.1519/00126548-200608000-00006.
